# 3β-Hydroxy-5β-hydroxy-B-norcholestane-6β-carboxaldehyde (SEC-B) Induces Proinflammatory Activation of Human Endothelial Cells Associated with Nitric Oxide Production and Endothelial Nitric Oxide Synthase/Caveolin-1 Dysregulation

**DOI:** 10.3390/antiox11061148

**Published:** 2022-06-10

**Authors:** Maria Gemma Nasoni, Serena Benedetti, Rita Crinelli, Francesco Palma, Barbara Canonico, Francesca Monittola, Chiara Zerbinati, Luigi Iuliano, Francesca Luchetti

**Affiliations:** 1 Department of Biomolecular Sciences, University of Urbino Carlo Bo, 61029 Urbino, Italy; maria.nasoni@uniurb.it (M.G.N.); serena.benedetti@uniurb.it (S.B.); rita.crinelli@uniurb.it (R.C.); francesco.palma@uniurb.it (F.P.); barbara.canonico@uniurb.it (B.C.); f.monittola@campus.uniurb.it (F.M.); 2 Department of Medico-Surgical Sciences and Biotechnology, Sapienza University of Rome, 04100 Latina, Italy; chiara.zerbinati@uniroma1.it (C.Z.); luigi.iuliano@uniroma1.it (L.I.); 3 UOC of Internal Medicine, Sapienza University of Rome and ICOT Hospital, 04100 Latina, Italy

**Keywords:** SEC-B, endothelial dysfunction, oxidative stress, inflammation, nitric oxide, eNOS, Caveolin-1, IL-6, TWEAK

## Abstract

Oxysterols are a family of 27-carbon cholesterol oxidation derivatives found in low-density lipoproteins (LDLs) and atherosclerotic plaques where they trigger several biological responses involved in the initiation and progression of atherosclerosis. Several pieces of evidence suggest that oxysterols contribute to endothelial dysfunction (ED) due to their ability to alter membrane fluidity and cell permeability leading to inflammation, oxidative stress and apoptosis. The present study aimed to investigate the molecular events occurring in human microvascular endothelial cells (HMEC-1) in response to autoxidation-generated 3β-hydroxy-5β-hydroxy-B-norcholestane-6β-carboxaldehyde (SEC-B) exposure. Our results highlight that SEC-B rapidly activates HMEC-1 by inducing oxidative stress, nitric oxide (NO) production and pro-inflammatory cytokine release. Exposure to SEC-B up to 24 h results in persistent accumulation of the vasodilator NO paralleled by an upregulation of the endothelial nitric oxide synthase (eNOS) enzyme and downregulation of Caveolin-1 (Cav-1) protein levels. Moreover, reduced expression and extracellular release of the vasoconstrictor factor endothelin-1 (ET-1) are observed. Furthermore, SEC-B stimulates the expression of the cytokines interleukin-6 (IL-6) and tumor necrosis factor-like weak inducer of apoptosis (TWEAK). This proinflammatory state leads to increased monocyte recruitment on activated HMEC-1 cells. Our findings add new knowledge on the role of SEC-B in ED and further support its potential implication in atherosclerosis.

## 1. Introduction

The endothelium is the thin monolayer of endothelial cells (ECs) lining the lumen of all blood vessels. ECs form an anti-inflammatory and anticoagulatory surface under normal circumstances and function as a selective barrier between the blood and other tissues [1]. Mechanosensors on ECs detect shear stress and transduce it into biochemical signals to trigger vascular adaptive responses. Among the various shear-induced signaling molecules, reactive oxygen species (ROS) and nitric oxide (NO) have been implicated in vascular homeostasis and diseases [2]. Indeed, oxidative stress and inflammation have been recognized as partners in crime in endothelial activation and dysfunction (ED), two crucial events driving the development and progression of the atherosclerotic plaque.

NO is a gaseous free radical synthesized by NO synthases (NOSs) starting from *_L_*-arginine, NADPH and oxygen that can exert physiologically multifaceted functions. NO is primarily identified as the most important vasoactive mediator produced by ECs; it acts as a powerful endogenous vasodilator, counterbalancing the effect of the vasoconstrictor factor endothelin-1 (ET-1). NO release is an important mechanism for ECs to protect the vasculature, and ED is commonly characterized by loss of NO bioavailability. NO plays critical roles in endothelial cell proliferation, leukocyte adhesion and angiogenesis. Nevertheless, NO is a highly reactive molecule, and its accumulation is potentially toxic and implicated in a wide range of pathological processes. Thus, the role of NO is not straightforward but mainly related to its “chemistry” and its cellular concentration [3]. NO may exert its adverse effects by interacting directly with biological targets or indirectly through the formation of *S*-nitrosothiols (RSNO) species. Several data indicate that the overproduction of NO and NOS is also responsible for inflammatory myopathies and muscle damage [4].

Caveolae are specialized plasma membrane domains containing the integral membrane protein caveolin (Cav) [5,6]. Cav-1 is the most abundant protein associated with caveolae; it is required for caveolae formation in non-muscle cells. Cav-1 is highly expressed in ECs, where the direct binding of endothelial NOS (eNOS) to the scaffolding domain of Cav-1 holds eNOS inactive [7].

Oxysterols are cholesterol oxidation products present in oxidized-low density lipoproteins (ox-LDLs) that regulate many biological processes and exert several biochemical effects of potential pathophysiological relevance. A growing body of evidence highlights that oxysterols are involved in cell proliferation and metabolism and exert proinflammatory and proapoptotic effects [8,9,10]. However, the effects of oxysterols on NO production and Cav-1 modulation are not totally understood.

Several papers have demonstrated that major components of the vascular wall, including ECs, vascular smooth muscle cells and fibroblasts, are important sources of cytokines. Interleukin-6 (IL-6) can stimulate endothelial expression of adhesion molecules, including intercellular adhesion molecule-1 (ICAM-1), vascular adhesion molecule-1 (VCAM-1) as well as E-selectin, thereby enhancing immune cell adherence and extravasation into the vascular wall [11]. Harada et al. have reported that TNF-like weak inducer of apoptosis (TWEAK), an inflammatory cytokine belonging to the tumor necrosis factor superfamily (TNF), is able to up-regulate the cell surface expression of ICAM-1 and E-selectin and to induce the secretion of interleukin-8 (IL-8) and C–C motif chemokine ligand 2 (CCL2) in human umbilical vein endothelial cells (HUVECs) [12]. Elevated levels of TWEAK have been detected in human atherosclerotic plaques [13], where it regulates cell growth, apoptosis and inflammation via activation of fibroblast growth factor-inducible 14 (Fn14) [14].

In this scenario, we sought to evaluate the ability of 3β-hydroxy-5β-hydroxy-B-norcholestane-6β-carboxaldehyde (SEC-B)—an autoxidation product of cholesterol found in atherosclerotic plaque—to dysregulate NO production and induce cytokine release, which are both known to be involved in endothelial activation and dysfunction.

## 2. Materials and Methods

### 2.1. Cell Culture and Treatments

A cell culture of human microvascular endothelial cells, HMEC-1 (ATCC, No CRL-3243), was obtained from the American Type Culture Collection (Teddington, UK). Cells were grown in MCDB-131 supplemented with 10% fetal bovine serum (FBS), 1% *_L_*-glutamine, 1% penicillin/streptomycin, 10 mM Hepes, 10 ng/mL Epidermal Growth Factor (EGF) and 1 µg/mL hydrocortisone at 37 °C in a humidified 5% CO_2_ incubator. 3β-hydroxy-5β-hydroxy-B-norcholestane-6β-carboxaldehyde (SEC-B) was synthesized by cholesterol ozonation [15] and was dissolved in ethanol. In order to evaluate the effect of SEC-B on HMEC-1, cells were incubated with SEC-B 20 µM resuspended in ethanol (<0.1% f.c.). For eNOS inhibition, HMEC-1 cells were pre-treated with L-NAME (100 and 500 μmol/L) (N5751; Sigma, St. Louis, MO, USA) for 1 h and then incubated with SEC-B for 4 and 24 h in complete medium.

### 2.2. WST-8 Cell Viability Assay

The effect of SEC-B on HMEC-1 cell viability was analyzed by WST-8 reagent [2-(2-methoxy-4-nitrophenyl)-3-(4-nitrophenyl)-5-(2,4-disulfophenyl)-2H-tetrazolium, monosodium salt] (Sigma-Aldrich, Milan, Italy). The assay was based on the cleavage of the tetrazolium salt WST-8 by cellular dehydrogenases in viable cells. Briefly, cells (5000/well) were incubated in clear 96-well plates with SEC-B for 24, 48 and 72 h. After incubation, WST-8 (1:10 final dilution) was added to each well, and cells were further incubated at 37 °C up to 4 h. Color development was monitored at 450 nm in a multiwell plate reader (ThermoFisher Scientific, Milan, Italy).

### 2.3. Wound Healing Assay (Scratch Assay)

The migratory and proliferative behavior of HMEC-1 was assayed by the wound healing assay. Cells were seeded in a 6-well plate at a density 4 ×10^5^ cells/dish, and after 24 h of adhesion, the cells were in a confluent cell monolayer (95–100%). Using a p200 pipette tip, a straight scratch was made, simulating a wound. Next, debris and non-attached cells were removed by washing the cell layer twice with 1 ml of sterile 1X PBS and then was replaced with the medium and the treatment. The migration rate into the “wound area” was observed and acquired after 3, 6 and 24 h under a phase-contrast microscope using the 10× objective with data acquisition software (Nikon ECLIPSE TS100, software NIS-Elements F, Nikon Europe BV, Amsterdam, Netherlands). The closure of the wound was calculated as follows: wound closure (%) = (A0 − An)/A0 × 100, where A0 represents the initial wound area and An the remaining area of the wound at the metering point.

### 2.4. Intracellular ROS Evaluation

Intracellular ROS were analyzed in HMEC-1 by 2′,7′-dichlorofluorescein diacetate (DCFH-DA, Sigma-Aldrich, Milan, Italy), which is a cell-permeable non-fluorescent probe that turns to highly fluorescent 2′,7′-dichlorofluorescein (DCF) upon oxidation. Briefly, cells (5000/well) in black 96-well plates were incubated with DCFH-DA (5 µM) for 30 min at 37 °C. After excess probe removal, cells were treated with SEC-B, and the fluorescence emission upon probe oxidation was monitored for 4 h at ex/em 485/520 nm in a FluoStar Optima (BMG Labtech, Ortenberg, Germany) multiwell plate reader. For microscopy analysis, cells were seeded at 1 × 105 cells/well in 6-well plates and, after SEC-B treatment, incubated with DCFH-DA (5 µM) for 30 min at 37 °C. Fluorescent images were observed and captured using a digital camera-attached fluorescence microscope with data acquisition software (Nikon ECLIPSE TS100, software NIS-Elements F, Nikon Europe BV, Amsterdam, Netherlands).

### 2.5. Measurement of NO Production

NO production was quantified intracellularly in HMEC-1 after stimulation with SEC-B by using the 4,5-diaminofluorescein (DAF-FM) diacetate (ThermoFisher, Milan, Italy) cell-permeable probe. DAF-FM diacetate is hydrolyzed to cell impermeable DAF-FM by intracellular esterases and reacts with NO and O^2^ to give the fluorescent DAF-FM. Briefly, cells (5000/well) were incubated in black 96-well plates and were loaded with 5 µM DAF-FM diacetate for 30 min at 37 °C. DAF-FM fluorescence was measured within cells at 495 nm excitation and 515 nm emission in the FluoStar Optima (BMG Labtech, Ortenberg, Germany) multiwell plate reader. For microscopy analysis, cells were seeded at 1 × 10^5^ cells/well in 6-well plates and, after SEC-B treatment, incubated with DAF-FM diacetate 5 µM for 30 min at 37 °C. Fluorescent images were observed and captured using a digital camera-attached fluorescence microscope with data acquisition software (Nikon ECLIPSE TS100, software NIS-Elements F, Nikon Europe BV, Amsterdam, Netherlands).

### 2.6. ELISA Immunoassay

Supernatants of confluent monolayers of HMEC-1 cells treated for 4 and 24 h with 20 µM SEC-B were centrifuged at 1000× *g* for 10 min, collected and stored at −80 °C until assayed. Soluble ET-1, TWEAK and IL-6 levels from supernatants were assessed by a specific immunoassay.

ET-1 and IL-6 kits were from R&D Systems (Abingdon, UK) (detection limit 0.087 pg/mL, CV intra-assay 4.0%, CV inter-assay 7.6% for ET-1; detection limit 0.70 pg/mL, CV intra-assay 4.4%, CV inter-assay 3.7% for IL-6). The TWEAK kit was from Bender MedSystems GmbH (Vienna, Austria) (detection limit 9.7 pg/mL, CV intra-assay 7.9%, CV inter-assay 9.2%).

### 2.7. Cell Extracts and Western Immunoblotting Analysis

Cells were lysed in sodium dodecyl sulfate (SDS) buffer (50 mM Tris-HCl, pH 7.8, 0.25 M sucrose, 2% (*w*/*v*) SDS, 10 mM *N*-ethylmaleimide (NEM), supplemented with protease and phosphatase inhibitors), and whole extracts were immediately heated at 100 °C. Samples were then sonicated at 70 Watt for 40 s to reduce sample viscosity and centrifuged at 12,000× *g* to remove debris. Protein concentration was determined in the supernatant by the Lowry assay using bovine serum albumin as a reference standard. Proteins were then resolved on SDS polyacrylamide gels, blotted onto PVDF membranes and stained with A44717 no-stain protein labeling reagent (ThermoFisher Scientific). The reagent enabled detection of all proteins on the membrane post transfer. Since the signal intensity was in the linear range with the protein load over a wide range of protein concentrations, it could be used as the loading control. Blots were then probed with the following antibodies: anti-Cav-1 (D46G3, #3267), anti-[P]eNOS (Ser1177, #9571) and anti-eNOS (49G3, #9586) obtained from Cell Signaling Technologies. Total protein and immune complex detection were performed using a ChemiDoc MP imaging system (BioRad, Hercules, CA, USA). Quantification of the immunoreactive bands and of the total protein content was performed with Image Lab software (Bio-Rad, Hercules, CA, USA). 

### 2.8. Caveolin-1 (Cav-1) Immunofluorescence

HMEC-1 cells were grown on 35 mm MatTek glass-bottom dishes (MatTek Corporation; density, 1 × 10^5^ cells/well). After treatment, cells were washed (2×) with PBS and fixed for 15 min with 4% (*v*/*v*) paraformaldehyde (pH 7.4) at room temperature. The cells were washed again (2×) with PBS and then permeabilized for 15 min with 0.1% Triton X-100 in PBS at room temperature. Next, the cells were washed (2×) with PBS and incubated for 60 min with the blocking solution (PBS containing BSA 2% *w*/*v*). The cells were then incubated overnight at 4 °C with the anti-Cav-1 antibody described above. After being washed (3×) with PBS, the cultures were incubated for 60 min with conjugated anti-rabbit secondary antibody (1:100). Subsequently, the cells were washed (3×) with PBS, and fluorescent images were captured by confocal microscopy (Leica Microsystem, Wetzlar, Germany).

### 2.9. Real-Time PCR

Total RNA was extracted from treated or untreated HMEC-1 cells using an RNeasy mini kit (Qiagen, Valencia, CA, USA) as per the manufacturer’s instructions. cDNA was synthesized using a SuperScript® First-Strand Synthesis System kit (Life Technologies, Monza, Italy). Amplification and detection of ICAM-1, IL-6 and β-actin were carried out using StepOnePlus equipment (Applied Biosystems, Monza, Italy) and MicroAmp Fast Optical (ThermoFisher, Milan, Italy) 96-well optical plates. The mixture contained, for each well, 10 µL of PowerUp™ SYBR™ Green Master Mix (Applied Biosystems™), 0.4 µM of each primer, 1 µL of cDNA previously diluted 1:5 and nuclease-free water to a total of 20 µL. The pairs of primers were *ICAM-1* (accession number: NM_000201): GCCGGCCAGCTTATACACAA and CAATCCCTCTCGTCCAGTCG; *IL-6* (accession number: NM_001371096.1): GAGAAAGGAGACATGTAACAAGAGT and GCGCAGAATGAGATGAGTTGT; and *β-actin* (accession number: NM_001101.5): GCGAGAAGATGACCCAGATC and GGATAGCACAGCCTGGATAG. Instead, qPCR of eNOS, ET-1, TWEAK and β-actin was performed using the TaqMan Fast Advanced Master Mix (Applied Biosystems). The total reaction volume (20 μL) consisted of the following: 1 μL cDNA diluted 1:5, 10 μL TaqMan Fast Advanced Master Mix, 1 μL of each TaqMan Gene Expression Assay (ThermoFisher, Milan, Italy) and 8 μL ultrapure DNase-free water. The TaqMan primers and probes used were *eNOS*: Hs01574665_m1; *ET-1*: Hs00174961_m1; *TWEAK*: Hs00356411_m1; and *β-actin*: Hs01060665_g1. The cycle parameters were as follows: UNG incubation at 50 °C for 2 min, polymerase activation at 95 °C for 20 s, denaturation at 95 °C for 1 s and then annealing and extension at 60 °C for 20 s. Relative fold changes in mRNA levels were calculated after normalization to β-actin using the comparative Ct method [16].

### 2.10. Flow-Cytometric Analysis of Supravital Propidium Iodide (PI) Staining and ICAM-1 Expression

HMEC-1 (5 × 10^4^ cells/well) were plated in 12-well plates for 72 h. At the end of the treatment, the cells were centrifuged and suspended in MCDB-131 containing 50 µg/mL propidium iodide for 30 min at room temperature in the dark [17]. To evaluate the surface expression of ICAM-1, HMEC-1 cells were treated for 24 h with SEC-B 20 µM and then incubated for 1 h with PE-conjugated mouse anti human ICAM-1 antibody (eBioscience, clone HA58). The cells were analyzed with a FACSCanto II flow cytometer (BD Biosciences, San Diego, CA, USA) using FACSDivaTM software (BD Biosciences, San Diego, CA, USA). Flow cytometry data were collected by accumulating at least 10,000 events for each tube, and mean fluorescence intensity (MFI) was measured as quantification of the amount of the ICAM-1 surface protein at a single cell level.

### 2.11. Cell Adhesion Assay

HMEC-1 cells were cultured at a density of 1 × 10^5^ cells/well on 6-well plates in MCDB-131 with 10% FBS. U937 cells were grown in RPMI-1640 medium containing 10% FBS and were labelled with 5 µM calcein-AM (Molecular Probes, Eugene, OR, USA) for 30 min at 37 °C. After labeling, cells were washed, and 5 × 10^5^ cells/well were seeded onto SEC-B-treated HMEC-1 monolayers for 24 h and incubated for 1 h at 37 °C and 5% CO_2_. Co-cultured cells were washed, and the images were obtained at 485 nm excitation and 538 nm emission using a digital camera-attached fluorescence microscope with data acquisition software (Nikon ECLIPSE TS100, software NIS-Elements F, Nikon Europe BV, Amsterdam, The Netherlands).

### 2.12. Statistical Analysis

Statistical analyses were performed using Prism version 5.00 (GraphPad Software, San Diego, CA, USA). Assays were carried out in triplicate, and the results were expressed as the mean values ± SD. Differences between samples were assessed by analysis of variance (ANOVA) with a Bonferroni post hoc test. The means of two groups were compared by using a *t*-test. The results were considered statistically significant when *p* < 0.05.

## 3. Results

### 3.1. Effect of SEC-B on HMEC-1 Cell Viability and Damage

**Figure 1:**
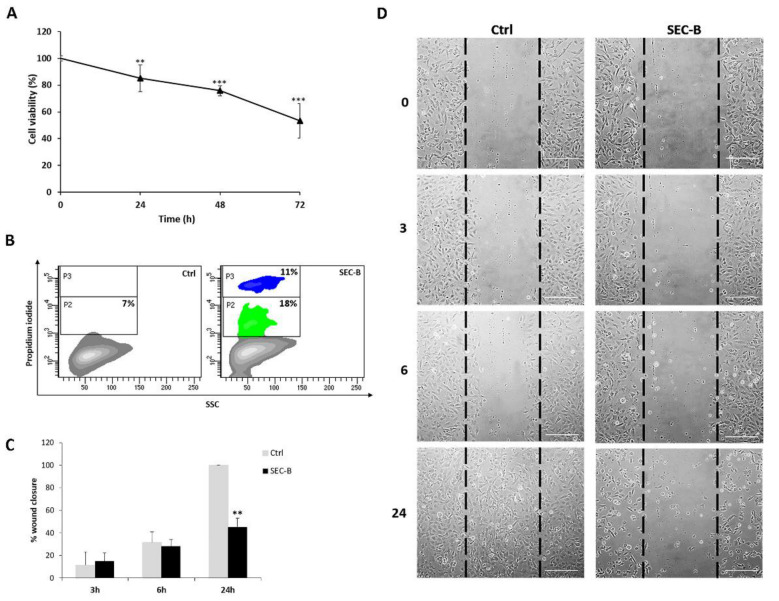
Effect of SEC-B on endothelial cell viability and proliferation. (**A**) Cell viability evaluation by WST-8 colorimetric assay upon 20 µM SEC-B administration for 24 h, 48 h and 72 h. (**B**) Representative supravital PI contour plots of treated HMEC-1 at 72 h showing apoptotic (green) and necrotic (blue) populations in HMEC-1-treated cells. (**C**) Quantitative analysis of the wound healing assay performed at 3 h, 6 h and 24 h after 20 µM SEC-B administration. Data are expressed as mean ± SD (*n* = 3). ** *p* < 0.01; *** *p* < 0.001 vs. untreated cells. (**D**) Representative images of the wound healing assay performed at 3 h, 6 h and 24 h after 20 µM SEC-B administration, as obtained by optical microscopy using the 10× objective. Scale bar: 200 µm.

The effect of SEC-B on cell viability was evaluated by WST-8 and propidium iodide assays. As shown in Figure 1A, cell treatment with 20 μM SEC-B resulted in a time-dependent decrease in cell viability. In particular, SEC-B at 24 h induced a significant reduction of cell viability (** *p* < 0.01 vs. control) that became more evident when the treatment was prolonged up to 72 h (*** *p* < 0.001 vs. control) with a sharp decrease of viable cells to 54%. Propidium iodide staining (Figure 1B) clearly demonstrated that the treatment with SEC-B significantly increased the number of apoptotic cells (green area identified as P2), with the concomitant appearance of a necrotic population (blue area identified as P3). To evaluate the ability of HMEC-1 to proliferate and repair damaged tissue, a wound healing assay was performed. Analysis of the wound healing assay showed that SEC-B treatment markedly reduced the motility of HMEC-1 cells up to 24 h of treatment, as determined by wound closure quantification (Figure 1C). The representative images captured during 24 h of SEC-B incubation show that SEC-B impaired the ability of ECs to close the wound scratch (Figure 1D).

### 3.2. SEC-B Increases Intracellular ROS Production

**Figure 2:**
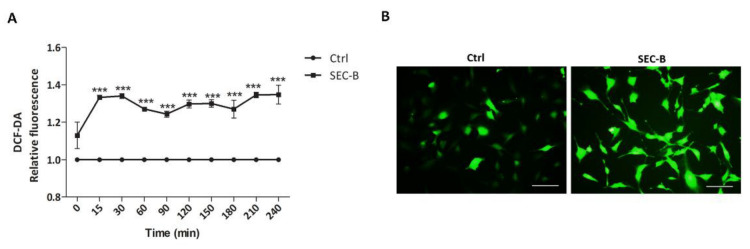
SEC-B induces ROS production in HMEC-1 cells. (**A**) Increased intracellular ROS levels in HMEC-1 cells within the first 4 h of SEC-B treatment. Data are expressed as a fold increase of DCF-DA fluorescence emission compared to control conditions. The mean ± SD (*n* = 3) is indicated. *** *p* < 0.001 vs. untreated cells. (**B**) Representative microscopy images of HMEC-1 labeled with DCF-DA probe after 4 h of SEC-B treatment. The images are obtained by optical fluorescence microscopy using the 20× objective. Scale bar: 100 µm.

Oxidative stress has been largely identified as one of the main factors involved in the pathogenesis of macrovascular diseases, playing a key role in the inflammatory response [18]. The effect of SEC-B on ROS production was assessed in DCF-DA-loaded HMEC-1 cells. Our data indicated that SEC-B increased the levels of ROS (+1.35-fold compared to untreated cells), which remain constantly elevated within the first 4 h of treatment (Figure 2A). Microscopic observation of SEC-B-treated HMEC-1 cells confirmed ROS production. In detail, weak and diffuse fluorescence was observed in the cytoplasm of control cells, whereas brilliant fluorescence was visible in all the cells after treatment with SEC-B (Figure 2B).

### 3.3. SEC-B Increases Intracellular NO Content and Modulates eNOS/Cav-1 Expression

**Figure 3:**
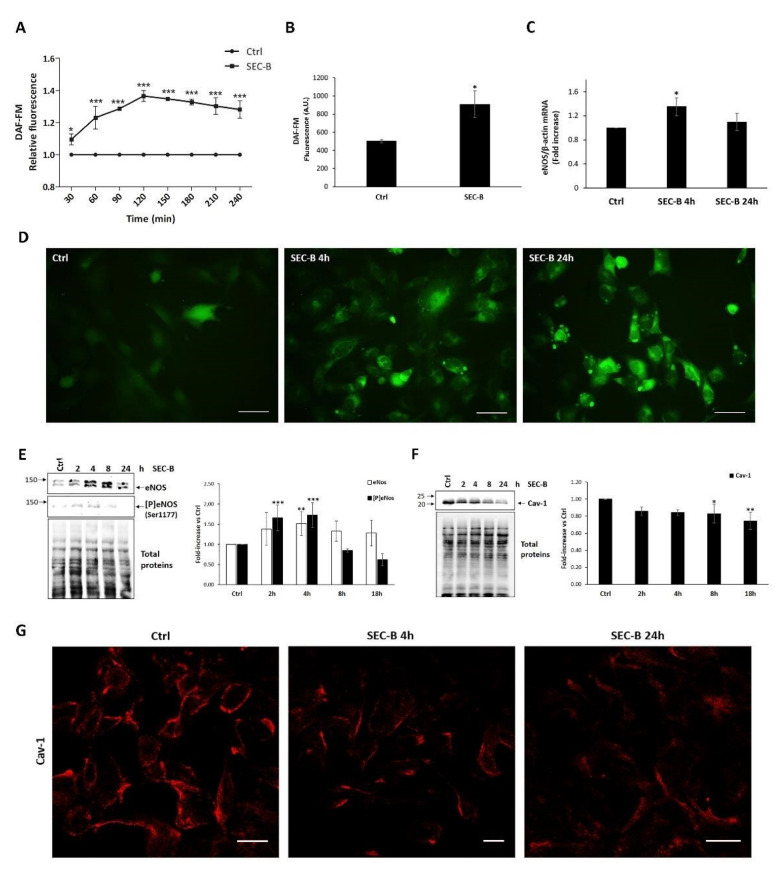
SEC-B induces NO accumulation and eNOS/Cav-1 modulation in HMEC-1 cells. (**A**) Intracellular NO increases during the first 4 h of SEC-B treatment. Data are expressed as a fold increase of DAF-FM fluorescence emission compared to control conditions. (**B**) Intracellular NO level after 24 h of SEC-B treatment. The DAF-FM fluorescence is reported as arbitrary units (A.U.). The means ± SD (*n* = 3) are indicated. (**C**) Real-time PCR of *eNOS* mRNA expression at 4 h and 24 h post-treatment. Data are normalized with β-actin. (**D**) Representative microscopy images of HMEC-1 labeled with DAF-FM probe after 4 h and 24 h of SEC-B treatment. The images are obtained by optical fluorescence microscopy using the 40× objective. Scale bar: 50 µm. (**E**,**F**) Western-immunoblotting analysis of eNOS, [P]eNOS (Ser1177) and Cav-1 levels in whole cell extracts. Then 20 μg of proteins were loaded onto 8% (*w*/*v*) SDS polyacrylamide gels for eNOS and [P]eNOS detection, while 5 μg of proteins were loaded onto 12% (*w*/*v*) SDS polyacrylamide gels for Cav-1 analysis. After blotting, total protein was stained on the membrane using the no-stain reagent as described in Materials and Methods, and then protein targets were detected using specific antibodies. The position of molecular weight markers (kDa) is shown on the left. Images were acquired in a ChemiDoc system, and signal intensity relative to immunoreactive bands and total protein content were quantified with Image Lab software. Values were normalized on total proteins and expressed as fold-increase vs. the value obtained in untreated cells set as 1. Bars represent the mean ± SD (*n* = 3). (**G**) Confocal images of Cav-1 immunostaining in HMEC-1-treated cells after 4 h and 24 h of SEC-B administration. Scale bar: 20 µm. Data are expressed as mean ± SD (*n* = 3). * *p* < 0.05; ** *p* < 0.01; *** *p* < 0.001 vs. untreated cells.

In ECs, NO is a key factor implicated in the maintenance of vascular homeostasis [18]. Thus, we next sought to monitor intracellular NO production in response to SEC-B administration. To this end, untreated (Ctrl) and SEC-B-treated cells were labeled with the NO DAF-FM probe. At early time points, we found that SEC-B induced an increase of NO content, which reached a peak at 2 h (+1.4-fold compared to untreated cells; *** *p* < 0.01) (Figure 3A) and remained elevated up to 24 h of incubation (* *p* < 0.05) (Figure 3B). Accumulation of NO was also confirmed by microscope observation of DAF-FM-labeled cells (Figure 3D). In cardiovascular diseases, increased/decreased NO bioavailability has been found associated with dysregulation of eNOS [19]. In our experimental conditions, *eNOS* mRNA expression was indeed significantly upregulated by SEC-B treatment at 4 h of incubation (Figure 3C). Between 2 h and 4 h, increased protein and Ser1177 phosphorylated eNOS levels were detected by immunoblotting analysis using specific antibodies (Figure 3E). In addition to the 140 kDa immunoreactive band, the anti eNOS antibody recognized a second protein band migrating at about 150 kDa and having a similar trend to the former. At the moment we do not have a reasonable explanation for this observation, although in myocytes it has been demonstrated that eNOS can be translated as a cytosolic 150 kDa isoform that is subsequently processed to the palmitoylated 135–140 kDa isoform found at the membrane [20].

Cav-1 is highly expressed in ECs where the direct binding with eNOS is able to inactivate the eNOS enzyme [7]. To investigate Cav-1 modulation upon SEC-B treatment, we analyzed Cav-1 protein expression by immunoblotting and confocal microscopy. The results shown in Figure 3F demonstrate that SEC-B induces a progressive reduction of Cav-1 protein levels. This result was confirmed by confocal immunostaining, where a significant decrease of the membrane punctate labeling of the protein was observed in SEC-B-treated compared to Ctrl cells (Figure 3G). Therefore, the loss of Cav-1 could explain why NO levels increase at 24 h despite eNOS mRNA and protein levels tending to return to basal levels.

### 3.4. SEC-B Down-Regulates ET-1 and Up-Regulate Pro-Inflammatory Cytokines IL-6 and TWEAK Expression

**Figure 4:**
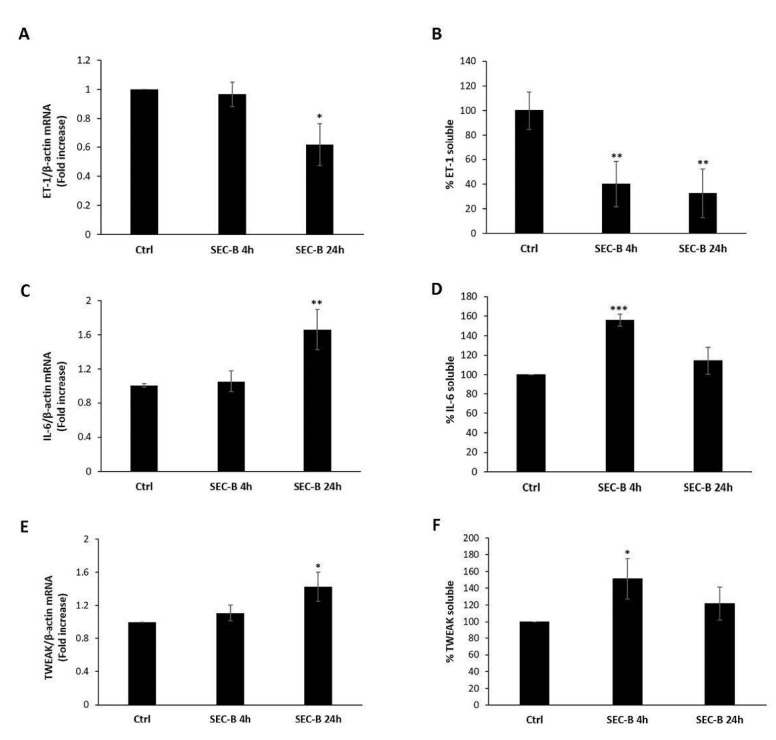
Effect of SEC-B on ET-1 and cytokine expression and secretion in HMEC-1 cells. Real-time PCR analysis of *ET-1* (**A**), *IL-6* (**C**) and *TWEAK* (**E**) mRNA expression at 4 h and 24 h post-treatment in cells treated with SEC-B. Data are normalized with β-actin mRNA. Quantitation of soluble ET-1 (**B**), IL-6 (**D**) and TWEAK (**F**) in the culture medium after 4 h and 24 h of SEC-B treatment by ELISA. Concentrations of soluble biomarkers (calculated as pg/mL) are expressed as % vs. untreated cells (Ctrl, set to 100%). Data are expressed as mean ± SD (*n* = 3). * *p* < 0.05; ** *p* < 0.01; *** *p* < 0.001 vs. untreated cells.

To further characterize the molecular events involved in SEC-B-induced endothelial activation/dysfunction, the expression and secretion of ET-1 and of some proinflammatory factors, such as IL-6 and TWEAK, were investigated. ET-1 is a vasoconstrictor secreted by ECs, which acts as the natural counterpart of the vasodilator NO [21]. Our results showed that SEC-B led to a significant decrease of *ET-1* mRNA expression at 24 h of treatment (* *p* < 0.05) (Figure 4A). Moreover, SEC-B exposure limited ET-1 secretion as demonstrated by the marked down-regulation of ET-1 protein levels released in the culture medium within 24 h of treatment (Figure 4B). On the contrary, HMEC-1-treated cells up-regulated *IL-6* mRNA expression (Figure 4C) and IL-6 protein secretion at 4 h of treatment (Figure 4D). The same trend was found for TWEAK, a cytokine involved in endothelium remodeling under chronic and acute inflammation [13]. Real-time PCR analysis showed up-regulation of *TWEAK* mRNA expression and protein secretion within 24 h of treatment (* *p* < 0.05 vs. untreated cells, Figure 4E,F). In detail, we found that SEC-B induced the release of soluble TWEAK in the medium after 4 h of treatment with an increase of 51% of TWEAK compared to the untreated condition (* *p* < 0.05 vs. untreated cells, Figure 4F).

### 3.5. SEC-B Enhances ICAM-1 Expression and Adhesion of U937 Cells

**Figure 5:**
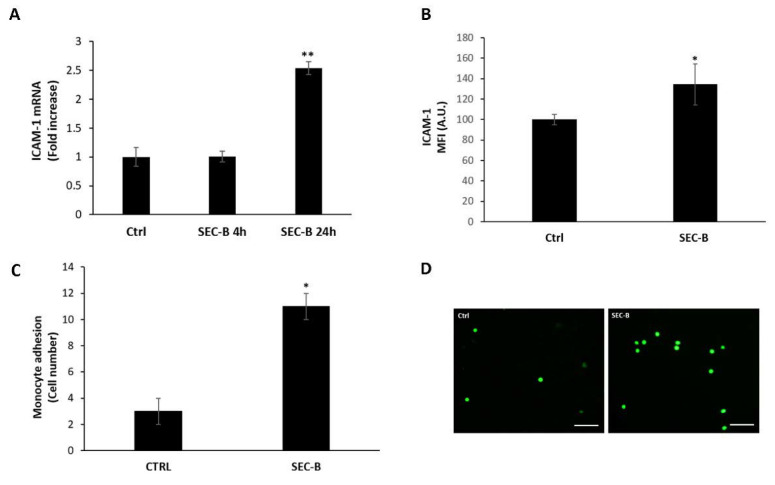
SEC-B upregulates ICAM-1 expression and promotes adhesion of U937 cells. (**A**) Real-time PCR analysis of *ICAM-1* mRNA expression after 4 h and 24 h of SEC-B treatment. (**B**) Evaluation of ICAM-1 surface expression at 24 h of SEC-B treatment by flow cytometry. (**C**) Quantification of the number of bound U937 cells in six randomly selected images with 10× magnification. (**D**) Representative fluorescence images showing the effects of SEC-B on adhesion of calcein-AM-labeled U937 cells to HMEC-1 treated for 24 h. Scale bar: 200 µm. Data are expressed as mean ± SD (*n* = 3). * *p* < 0.05; ** *p* < 0.01 vs. untreated cells.

It has been established that NO production and secretion of proinflammatory cytokines, in particular IL-6 and TWEAK, are accompanied by changes in the expression of adhesion molecules, which, in turn, promote leukocyte adhesion to the endothelium [22,23]. Our data demonstrated that SEC-B is able to upregulate *ICAM-1* mRNA expression (Figure 5A) with a significant up-regulation of ICAM-1 surface protein expression after 24 h of treatment (Figure 5B). Given the well-known role of ICAM-1 as an endothelial cell adhesion molecule mediating leukocyte recruitment to the endothelial tissue, we evaluated whether SEC-B was able to stimulate monocyte adhesion on HMEC-1. Cell adhesion was evaluated by quantification of the adherent calcein-labeled U937 cells to HMEC-1 (Figure 5C). In untreated cells a slight binding of U937 to HMEC-1 was observed, whereas SEC-B-treatment significantly increased the adhesion of U937 cells to HMEC-1 (Figure 5C,D).

### 3.6. Effect of NO Inhibition on SEC-B-Induced HMEC-1 Cell Dysfunction

**Figure 6:**
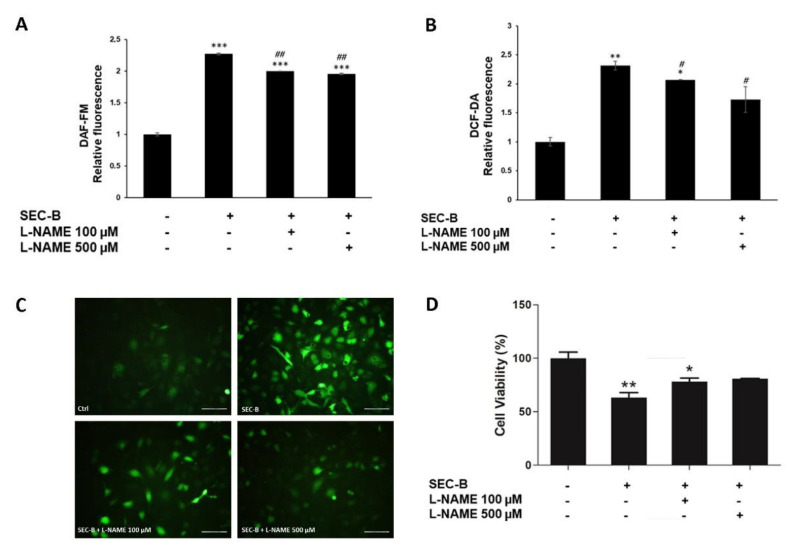
Inhibition of NOS decreases NO and ROS production and increases HMEC-1 cell survival. (**A**) Pre-treatment with L-NAME (100 µM and 500 µM) decreases intracellular NO levels in HMEC-1 cells after 4 h of SEC-B administration. (**B**) Pre-treatment with L-NAME decreases intracellular ROS levels in HMEC-1 cells after 4 h of SEC-B administration. (**C**) Representative microscopy images of HMEC-1 labeled with DCF-DA probe after 1 h of pre-treatment with L-NAME followed by 4 h of SEC-B treatment. The images are obtained by optical fluorescence microscopy using the 20× objective. Scale bar: 100 µm. (**D**) Cell viability evaluation after 1 h of pre-treatment with L-NAME followed by 24 h of SEC-B treatment. Data are expressed as mean ± SD (*n* = 3). * *p* < 0.05, ** *p* < 0.01, *** *p* < 0.001 vs. untreated cells; # *p* < 0.05, ## *p* < 0.01 vs. SEC-B-treated cells.

To investigate the role of NO in mediating SEC-B-induced HMEC dysfunction, we next performed experiments in the presence of the NOS antagonist L-NAME. Pre-treatment with L-NAME reduced both NO and ROS levels (## *p* < 0.001; # *p* < 0.005, respectively) in HMEC-1 stimulated with SEC-B (Figure 6A,B). The images of microscopy confirmed the dampening of DCF-DA fluorescence in the presence of L-NAME (Figure 6C). Under these experimental conditions, an increase of 17% of cell survival if compared to SEC-B-treated cells alone was also observed, highlighting that NO and ROS are among the mediators of cell dysfunction and death induced by SEC-B (Figure 6D).

**Figure 7:**
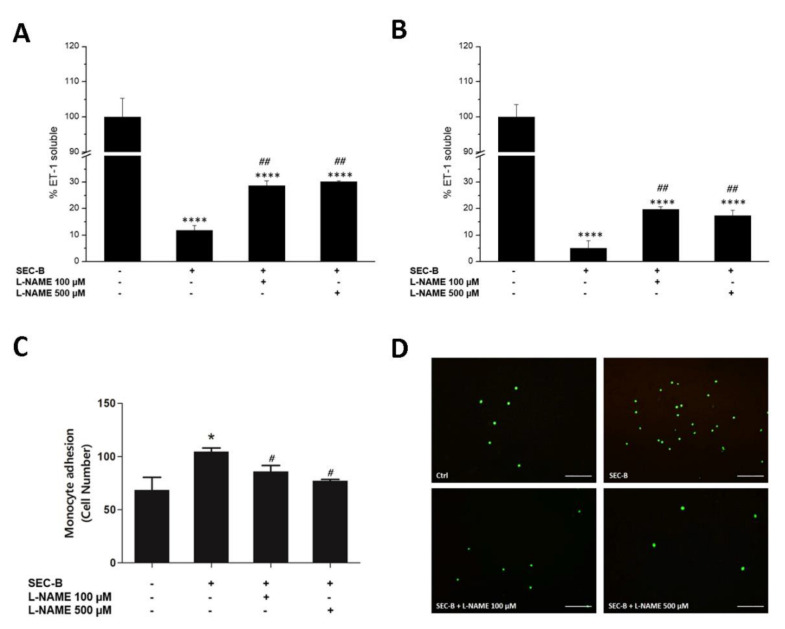
Inhibition of NOS decreases HMEC-1 cell activation. Pre-treatment with L-NAME (100 µM and 500 µM) increased soluble ET-1 levels in HMEC-1 cells after 4 h (**A**) and 24 h (**B**) of SEC-B administration. (**C**) Quantification of the number of bound U937 cells in six randomly selected images with 10× magnification. (**D**) Representative fluorescence images showing the effects of L-NAME on adhesion of calcein-AM-labeled U937 cells to HMEC-1 treated for 24 h with SEC-B. The images are obtained by optical fluorescence microscopy using the 10× objective Scale bar: 200 µm. Data are expressed as mean ± SD (*n* = 3). * *p* < 0.05, **** *p* < 0.0001 vs. untreated cells; # *p* < 0.05, ## *p* < 0.01 vs. SEC-B-treated cells.

As demonstrated above, SEC-B induced downregulation of ET-1 expression and secretion, an effect that can be associated with eNOS dysregulation and NO overproduction. Therefore, the release of ET-1 in response to L-NAME pre-treatment was next investigated. Our findings demonstrated a significant increase of ET-1 secretion at both 4 h and 24 h when cells were pre-treated with L-NAME before incubation with SEC-B (Figure 7A,B). Moreover, the adhesion assay confirmed that inhibition of NO production prevents endothelial activation, as demonstrated by a dose-dependent decrease of adherent U937 cells to HMEC-1 after L-NAME pre-treatment (# *p* < 0.05, Figure 7C,D). 

## 4. Discussion

ED is a pathological condition characterized by reduced vasodilation, pro-oxidative state and procoagulant activity. ED has been identified as the main event in the pathogenesis of macrovascular diseases, including atherosclerosis [24,25].

Among proatherogenic factors, oxysterols were recently identified as potential candidates in atheroma formation due to their proinflammatory and proapoptotic properties. Because several papers have documented a large amount of oxysterols in atherosclerotic plaques, the presence of these compounds has been related to the onset and progression of this pathology [8]. 

To date, the mechanisms through which oxysterols may impair endothelial function, thus promoting atherosclerosis, remain unclear. 

Data reported in this study highlight that 3β-hydroxy-5β-hydroxy-B-norcholestane-6β-carboxaldehyde (SEC-B) is able to cause up-regulation of eNOS protein and phospho protein levels in HMEC-1 cells, overproduction of NO as well as production of ROS.

Several papers suggest that oxidative stress can have multiple effects on NO signaling [26]. In particular, ROS can modulate eNOS activity and, on the other hand, eNOS itself may produce ROS when this enzyme is uncoupled. This interplay is also evident in our experimental conditions where the pre-treatment with L-NAME affects ROS production and ET-1 release. 

Furthermore, eNOS activation was accompanied by a significant reduction of Cav-1 protein expression. This finding is in agreement with the observation of Fielding et al. that oxysterols decrease Cav-1 mRNA levels in fibroblasts [27]. It is generally accepted that Cav-1, a plasma membrane-associated scaffolding protein, represses eNOS activity in ECs by direct sequestration, thus limiting the accessibility of Ca2+-induced calmodulin binding to eNOS [28,29]. Thus, reduction of Cav-1 protein levels at the plasma membrane may represent a second mechanism through which SEC-B may increase NO production in HMEC-1 cells. 

Increased NO production by eNOS activation in ECs modulates various cellular processes essential for endothelial integrity; conversely, perturbation of NO regulation contributes to several pathological states [3,30,31]. Some studies have reported that cholesterol/oxysterols can impair NO production through a variety of mechanisms, including decreased eNOS expression, reduced eNOS dimer formation or reduced eNOS substrate availability [32,33]. Kubes et al. demonstrated for the first time that inhibition of eNOS increases neutrophil adherence to mesenteric venules [22]. On the other hand, Yu and coworkers provided evidence that oxidized-LDLs at low concentration increase NO production and promote in vitro angiogenesis through the PI3K/Akt/eNOS pathway in human coronary artery endothelial cells [34]. In fact, the overproduction of NO has also been found responsible for the development of inflammatory pathologies [35,36,37,38]. Interestingly, SEC-B induced downregulation of ET-1 expression and secretion, an effect that can be associated with eNOS dysregulation and NO overproduction. Indeed, ET-1 has been shown to reduce eNOS expression and NO generation in fetal pulmonary artery endothelial cells [39]. In the literature, there is scarce evidence about the effects of oxysterols on ET-1 modulation. Different degrees of LDL oxidation have been shown to induce different effects on ET-1 regulation. In particular, He et al. demonstrated that extensively oxidized LDLs inhibit ET-1 secretion from cultured ECs. The authors speculated that inhibition of ET-1 release could exert a protective effect, reducing vessel wall tone near atherosclerotic lesions [40].

Moreover, it has been recently reported that the liver X receptor (LXR), a nuclear receptor regulated by oxysterols, downregulates ET-1 gene expression by interfering with the AP-1/NF-kB signaling pathways [41], providing a possible link between oxysterols and ET-1 expression. Several studies have highlighted that a persistent proinflammatory state in ED can ultimately lead to cell death [42,43]. Consistent with these findings, SEC-B treatment decreased cell viability with an impairment of the ability to proliferate and repair damaged tissue. On the other hand, pre-treatment with the NOS antagonist L-NAME increases survival of SEC-B-treated cells. During the early stages of atherosclerosis, ox-LDL accumulated in the intima induces the activation of both endothelial cells and vascular smooth muscle cells, leading to the expression and secretion of several proinflammatory cytokines, chemokines and adhesion molecules that recruit monocytes within the arterial wall [44]. Similarly to LDL, here we provide evidence that SEC-B is able to induce the expression of some cytokines implicated in several aspects of vascular inflammation, such as IL-6 and TWEAK and of the adhesion molecule ICAM-1 [45,46].

Since NO is classically described as a negative regulator of leukocyte adhesion, upregulation of ICAM-1 and increased U937 recruitment on HMEC-1 cells was a quite unexpected result. However, a recent study by Aguilar et al. demonstrated that TNF-α-activated eNOS signaling can increase leukocyte adhesion through ICAM-1 and the S-nitrosylation pathway [47,48,49]. In line with these data, our findings also demonstrated that overproduction of NO plays a key role in U937 cellular adhesion. Expression of adhesion molecules, including ICAM-1, VCAM-1 as well as E-selectin, can be also stimulated by IL-6 [11,50,51]. IL-6 is a primary mediator of the acute phase response; it is secreted by various cells, including ECs. IL-6 levels are increased in cardiovascular disease, including atherosclerosis and hypertension, where it is thought to promote alterations in vascular function and structure [52]. Multiple evidence indicates that increased ROS production occurs upstream of IL-6 expression and induction of endothelial permeability [52,53]. Indeed, several inflammatory cytokines affect the expression and activity of both eNOS and NADPH oxidase, thus influencing NO and superoxide levels and contributing to oxidative stress [54,55]. In the present study, we also demonstrate, for the first time, that the treatment with SEC-B is able to promote the expression and release of TWEAK in ECs. 

TWEAK is a cytokine belonging to the TNF superfamily that exists in two forms: a membrane bound form and a soluble one. Induction of TWEAK expression has frequently been reported in scenarios of tissue inflammation and damage [56]. Nowadays, there is little knowledge about the expression and secretion of this cytokine in ECs, whereas it is well-established that ECs are targets of TWEAK [57,58,59]. It has been reported that TWEAK enhances vascular and renal damage through the expression of NF-kB-regulated chemokines such as RANTES and MCP-1 [60]. Our findings highlight that oxysterols are able to induce early secretion of TWEAK coupled with increased expression, suggesting potential involvement in the development of atherosclerotic plaques. In fact, when TWEAK binds its receptor Fn14, it exerts several adverse biological functions including dysfunction of ECs and inflammatory responses of monocytes/macrophages [13]. 

Overall, our results show that SEC-B induces ED, promoting EC inflammatory activation and eNOS/Cav-1 dysregulation, two events which may be tightly connected and that may both contribute to atherogenesis.

## 5. Conclusions

In conclusion, our findings demonstrate that SEC-B is able to induce overproduction of NO, promoting ED. Our study sheds light on the paradigm that NO inhibits leukocyte adhesion and inflammatory cytokine release, revealing this to be a key factor in the loss of vascular endothelial integrity.

Overall, this study provides novel insights into SEC-B-induced ED, highlighting its role in atherogenesis development.

## Data Availability

The data presented in this study are available in the article.
